# Comprehensive Characterization of the *FAT*s Gene Family in Maize: Phylogeny, Expression Patterns, and Regulatory Networks

**DOI:** 10.3390/genes16091035

**Published:** 2025-08-30

**Authors:** Yunlong Li, Shuai Hou, Yan Sun, Shujun Li, Minghao Sun, Baitao Guo, Luyao Wang, Quan Cai, Xin Li, Sinan Li, Jianguo Zhang

**Affiliations:** Heilongjiang Academy of Agricultural Sciences, Harbin 150086, China; 13945699869@163.com (Y.L.); shuai0304@126.com (S.H.); sunyan19850301@163.com (Y.S.); lshj_750425@163.com (S.L.); sunminghao_yg@yeah.net (M.S.); 18904650420@163.com (B.G.); grace19940530@126.com (L.W.); cq6539@163.com (Q.C.); maize_lee@163.com (X.L.); mrlee890323@163.com (S.L.)

**Keywords:** fatty acyl–ACP thioesterase, maize, fatty acid biosynthesis, gene expression

## Abstract

Background: Fatty acyl–ACP thioesterase (FAT) genes regulate fatty acid composition and content, yet the FAT family in maize has not been systematically characterized. Methods: Ten ZmFAT genes were identified from the maize genome and analyzed for gene structure, protein properties, phylogeny, collinearity, cis-acting elements, and predicted interactions. Transcriptome and qRT–PCR data were used to assess expression patterns during seed development. Results: The ten ZmFAT genes were grouped into two subfamilies (three ZmFATA and seven ZmFATB genes). Two pairs of collinear genes were detected within maize and one pair between maize and rice. Promoter analysis revealed light- and development-responsive elements. Two genes were functionally annotated in fatty acid biosynthesis, while five proteins exhibited interactions and 14 miRNAs were predicted to regulate ZmFAT genes. Expression analysis showed that ZmFATA1/2 and ZmFATB4/6/7 maintained high expression in both upper and lower seed parts, and qRT–PCR confirmed their gradual upregulation during seed development. Conclusion: This study provides the first comprehensive characterization of the maize ZmFAT family, offering insights into fatty acid metabolism and valuable genetic resources for improving maize oil composition.

## 1. Introduction

Fatty acid biosynthesis (FAS) is an indispensable fundamental metabolic pathway extensively involved in phospholipid synthesis, cell membrane assembly, cellular signal transduction, energy storage, and gene expression regulation [1,2]. In higher plants, fatty acid biosynthesis occurs in plastids [3]. Acetyl–CoA serves as the precursor for this pathway and is converted into malonyl–CoA by acetyl–CoA carboxylase, which provides the elongation substrate for fatty acid biosynthesis [4,5]. Subsequently, type II fatty acid synthase catalyzes successive condensation reactions between malonyl–CoA and acyl–ACP derivatives, elongating acyl chains by two carbons per cycle to produce 16– to 18–carbon saturated fatty acids [6]. Fatty acyl–ACP thioesterase (FAT) terminates fatty acid synthesis by hydrolyzing the thioester bond between the acyl group and ACP, thereby releasing free fatty acids and ACP [7]. The fatty acids are transported to the cytoplasm for further esterification to form acyl–CoA, which then undergoes fatty acid chain elongation and desaturation on the endoplasmic reticulum, incorporation into membrane lipids as phospholipids, and metabolism and storage through triacylglycerol synthesis [8,9,10]. FAT plays a crucial role in regulating lipid metabolism and storage.

FATs are plastid-targeted soluble enzymes encoded by nuclear genes [11]. Based on their amino acid sequences and substrate specificities, FATs are classified into two subfamilies: FATA and FATB [12]. The substrate specificity of FATs largely determines the chain length and degree of unsaturation of plant fatty acids. FATA preferentially acts on unsaturated acyl–ACP, exhibiting the highest activity toward 18:1–ACP. In contrast, FATB shows limited activity toward 18:1–ACP but exhibits high activity toward saturated acyl–ACPs [13,14]. The *FAT* genes family has been identified and characterized in the genomes of various plants including *Glycine max* [15], *Arachis hypogaea* [16], *Elaeis guineensis* [17], and others. Moreover, *FAT* genes have been cloned and functionally analyzed in plants such as *Brassica napus* [18], *Cinnamomum longepaniculatum* [19], *Helianthus annuus* [20], and cotton [21].

The substrate specificity of FAT determines fatty acid composition and content [21]. Disruption of the *AtFATB* gene resulted in a 40–50% reduction in total saturated fatty acid content in different tissues compared with wild-type Arabidopsis, with palmitic acid content significantly decreased [22]. In soybean, *GmFATs* determine the ratio and composition of saturated and unsaturated fatty acids. Mutations in *GmFATA1A* and *GmFATB* resulted in increased oleic acid content, while *GmFATB* mutation reduced palmitic acid content [15]. Fatty acid profiling of tobacco leaves transiently expressing *GhA-FatB3* or *GhD-FatB4* demonstrated that they have high substrate preference for 16:0-ACP and resulted in palmitic acid enrichment [21].

Maize (*Zea mays* L.) is widely cultivated worldwide [23]. Maize germ can be processed through a series of refining procedures to produce high-quality vegetable oil rich in linoleic acid, oleic acid, and palmitic acid [24]. Studies have shown that long-term consumption of corn oil reduces cholesterol absorption and synthesis to some extent [25], thereby slowing the development of atherosclerosis [26]. This study, *ZmFATs* were identified and characterized from maize, providing a foundation for the genetic improvement of maize oil composition and content.

## 2. Materials and Methods

### 2.1. Identification of FATs Family Members in Maize

The maize genome and its annotation files were downloaded from the Ensembl Plants database (Zm-B73-REFERENCE-NAM-5.0). A genome-wide search was performed using the HMM model of the acyl-ACP thioesterase N-terminal domain (PF01643) with a threshold set at E < 1 × 10^−5^. Sequences were manually submitted to the CDD (http://blast.ncbi.nlm.nih.gov, accessed on 20 December 2024), pfam (https://www.ebi.ac.uk/interpro/, accessed on 20 December 2024) and SMART (http://smart.embl-heidelberg.de/, accessed on 20 December 2024) databases to exclude genes with incomplete structures and lacking conserved domains [27,28]. Corresponding protein sequences were retrieved based on the identified genes. Sequences were submitted to the ExPASy database (http://web.expasy.org/compute_pi/, accessed on 20 December 2024) to calculate the physicochemical properties of the proteins [29].

### 2.2. Phylogenetic Analysis of ZmFATs Family Members

To classify FATs family members from maize, Arabidopsis, and soybean, their protein sequences were aligned using the ClustalW algorithm in MEGA (v7) software [30]. After sequence alignment, a phylogenetic tree was constructed using the neighbor-joining (NJ) method with 1000 bootstrap replicates to build a robust evolutionary tree. FATs from *Arabidopsis* and soybean served as references for the systematic classification of maize ZmFATs. We used EvolView-v3 (https://www.evolgenius.info/evolview-v3/, accessed on 21 December 2024) to optimize the visualization of the phylogenetic tree.

### 2.3. Gene Structure and Motif Analysis of ZmFATs Family

The online tool MEME Suite v1.1 (http://meme-suite.org/tools/meme, accessed on 22 December 2024) was used to analyze gene structure and conserved motifs [31]. The maximum number of motifs was set to 15. TBtools (v2.301) was used to visualize motifs, introns, and exons of genes [32].

### 2.4. Chromosomal Localization and Collinearity Analysis of Maize ZmFAT Genes

The MCScanX tool was used to analyze homologous relationships among *FAT* genes from maize, rice, and *Arabidopsis* [33]. TBTools was used to visualize gene positions on chromosomes [32]. Genes with collinear relationships were connected by lines.

### 2.5. Cis-Elements Analysis

The upstream 2000 bp sequence of each *ZmFAT* was extracted as its promoter sequence and submitted to the PlantCARE database (https://bioinformatics.psb.ugent.be/webtools/plantcare/html/, accessed on 25 December 2024) to predict *cis*-acting elements (CREs) in the promoter region [34]. The R ggplot2 package was used for visualization.

### 2.6. Functional Annotation Analysis

Gene Ontology (GO) functional annotation was performed using EggNOG database and clusterProfiler (v4.17.0) software package with pvalueCutoff = 0.05 and qvalueCutoff = 0.05. Functional annotation results were visualized using the R package ggplot2 (v3.5.2).

### 2.7. Protein Interaction Prediction

ZmFAT protein sequences were submitted to the STRING database (https://string-db.org/, accessed on 26 December 2024) to search and predict protein interaction relationships [35], and the protein interaction network was visualized using the R package ggplot2.

### 2.8. miRNA Interaction Analysis with Maize ZmFATs

To construct the miRNA–gene interaction network, *ZmFATs* CDS sequences were submitted to the psRNATarget website (https://www.zhaolab.org/psRNATarget/, accessed on 27 December 2024). Using the website’s default parameters, putative miRNAs targeting *ZmFAT* genes were predicted, and the results were visualized using Cytoscape (v3.10.3) [36].

### 2.9. Transcriptome Analysis

Transcriptome data from different developmental stages of maize seed upper (U) and lower (B) parts after pollination were downloaded from the NCBI website (https://www.ncbi.nlm.nih.gov/, accessed on 28 December 2024) (BioProject IDs: PRJNA1027494 and PRJNA1027500) to analyze the expression patterns of the *ZmFAT* family at different developmental stages of maize seeds (4, 5, 6, 8, 10, 12, 16, 20, 24, 28, 32 d). Salmon V1.10.3 software was used to quantify the expression of *ZmFATs* from the transcriptome data.

### 2.10. qRT–PCR Analysis

Maize inbred line B73 was planted in Harbin. Seeds from different developmental stages in the upper (U) and lower (B) parts were collected for qPCR analysis, with three independent biological replicates for each sample. RNA was extracted from maize seeds using the RNAqueous™ Micro Total RNA Isolation Kit (ThermoFisher, Waltham, MA, USA). Then, cDNA was synthesized from total RNA using the RevertAid™ First Strand cDNA Synthesis Kit (ThermoFisher, Waltham, MA, USA). Real-time qPCR analysis was performed on a Bio–Rad CFX96 Real-Time PCR Detection System (Bio–Rad, Hercules, CA, USA) with ChamQ Universal SYBR qPCR Master Mix (Vazyme, Nanjing, China). *ZmActin1* was used as the reference gene, and the primer sequences are listed in Table S1. The following qPCR protocol was used: denaturation at 95 °C for 3 min, followed by 40 cycles of 15 s at 95 °C and 20 s at 65 °C for amplification. Target genes were quantified using the 2^−ΔΔCt^ method [37].

### 2.11. Statistical Analysis

Experimental data are presented as means ± standard error from at least three independent biological replicates. *p*-values were calculated by one-way ANOVA method using SPSS ver. 25.0 (SPSS Inc., Chicago, IL, USA), with *p* < 0.05 representing significant differences. Data were visualized using GraphPad Prism 9.

## 3. Results

### 3.1. Identification and Characterization of Maize FAT Family Members

**Figure 1:**
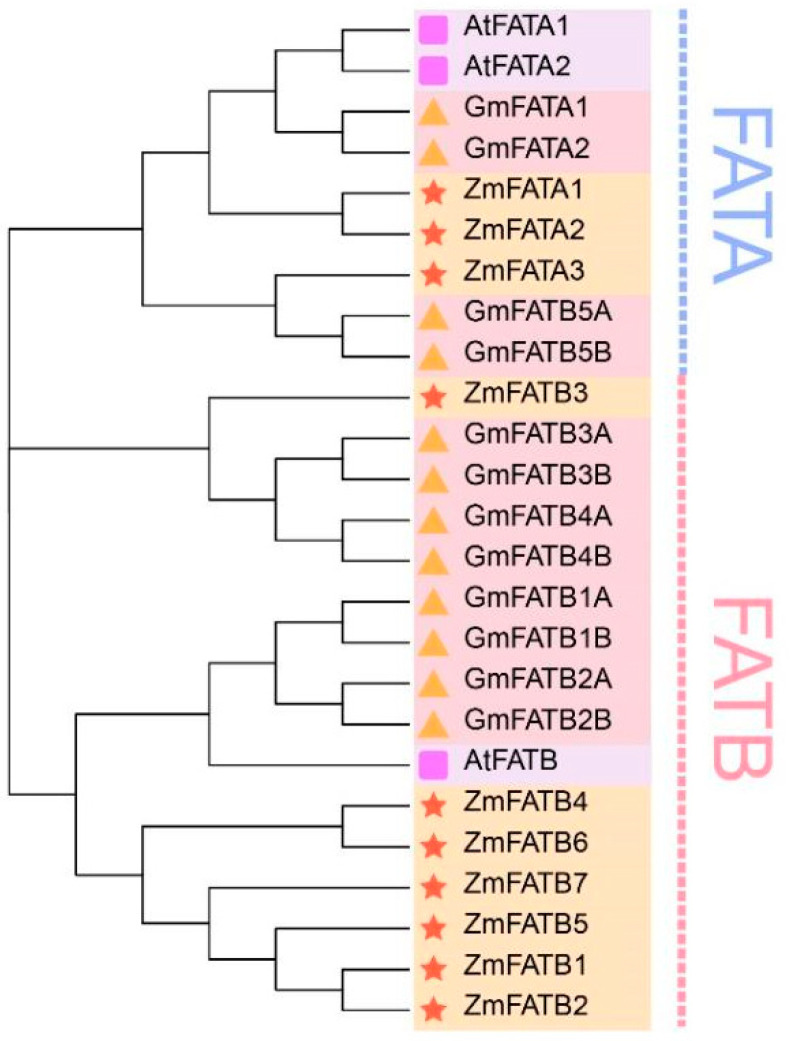
Phylogenetic tree of FATs proteins from maize, Arabidopsis, and soybean. The same shapes and background colors represent proteins from the same plant. Subgroups are marked with different colored text and dashed lines on the right side.

To identify the maize *FAT* gene family, we searched the maize genome using HMM search and BLASTP. All candidate sequences were subsequently verified by manual domain analysis with Pfam, SMART, and NCBI–CDD databases. Ten *FAT*s were identified. Based on their chromosomal positions and phylogenetic relationships, they were designated as Zm*FATA1*–*3* and Zm*FATB1*–*7* (Table 1 and Figure 1). The physicochemical properties of proteins encoded by *ZmFAT* genes were calculated (Table 1). ZmFATAs comprised 157–426 amino acids (aa) with molecular weights ranging from 16.96–47.67 kDa. Among ZmFATBs, ZmFATB5 had the fewest amino acids and lowest molecular weight (83 aa, 9.74 kDa). ZmFATB6 contain the most amino acids (434 aa) and had the highest molecular weight (47.76 kDa). Furthermore, the isoelectric point (pI) reflects the acidity and alkalinity of proteins. Only ZmFATB1 had a pI below 6 (5.4), indicating acidity. ZmFATA1–3 and ZmFATB2–7 all had pI values greater than 6 (6.37–10.29), indicating alkalinity. The grand average of hydropathicity (GRAVY) values were all < 0 (−0.75 to −0.08), suggesting that ZmFATs generally possess hydrophilic and soluble characteristics.

### 3.2. Phylogenetic Classification of the Maize FATs Family

Twelve GmFATs, 3 AtFATs, and the identified 10 ZmFATs were used to construct the phylogenetic tree. The phylogenetic tree was divided into 3 major clades. Three ZmFATAs, 2 AtFATAs, and 4 GmFATAs clustered in a common clade (FATA). FATBs comprised two clades. ZmFATB3 clustered with 4 GmFATBs in the same clade. The other clade contained 1 AtFATB, 4 GmFATBs, and 6 ZmFATBs.

### 3.3. Analysis of Conserved Motifs and Gene Structure

**Figure 2:**
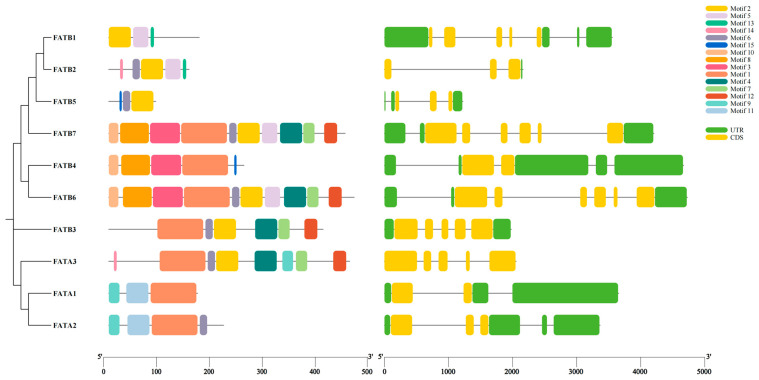
Phylogenetic tree, motif patterns, and gene structure of ZmFATs. Different colored blocks represent different motifs, UTRs, and CDS. Black lines in the right panel represent introns.

Gene structure potentially reflects gene function and evolutionary relationships [38]. Members of ZmFAT family were divided into 3 subgroups in the phylogenetic tree (Figure 2). ZmFATs contained 3–10 motifs. ZmFATB6/7 contained the most motifs (10), followed by ZmFATA3 (8), while ZmFATA1 and ZmFATB1/5 had the fewest motifs (3). Motif2 was present in all ZmFATs and represented the most conserved motif. Motif8/9 were most conserved in ZmFATAs. The gene structures of ZmFATs exhibited considerable variation. ZmFATB2 contained the fewest introns (3), while ZmFATB1/6/7 contained the most introns (6).

### 3.4. Chromosomal Localization and Collinearity Analysis

**Figure 3:**
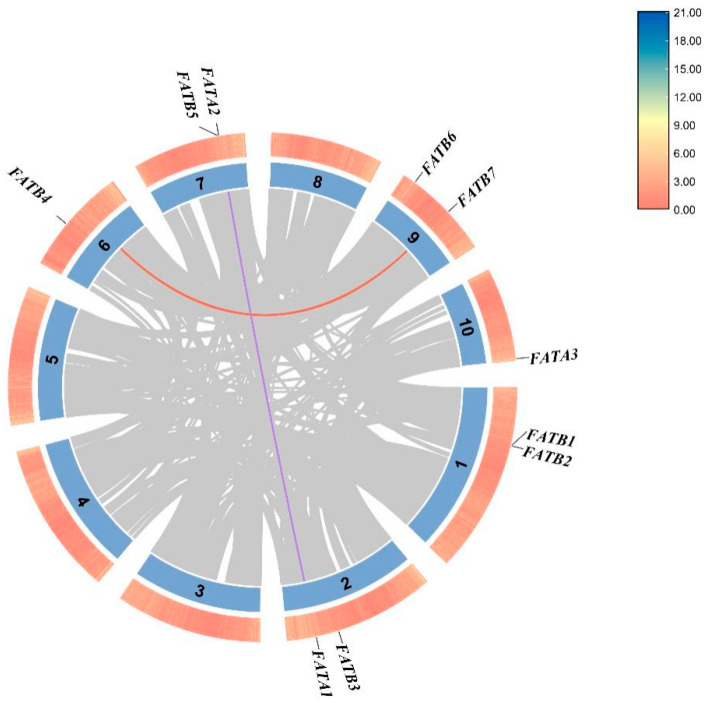
Collinearity among *ZmFATs* homologous genes. Gray lines as background represent the collinearity of genes across the whole genome, and colored lines are used to connect *ZmFAT*s pairs with collinear relationships.

**Figure 4:**
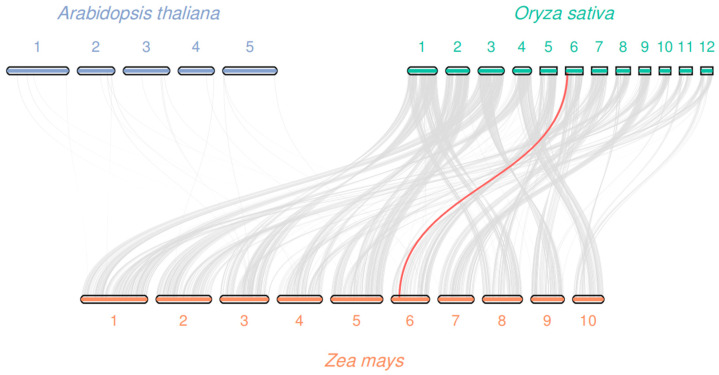
Collinearity among *FAT*s between maize and *Arabidopsis*/rice. Gray lines as background connect collinear genes in genomes of different species, and red lines connect *FAT*s gene pairs with collinearity between different species.

*ZmFAT*s were distributed on 6 chromosomes (chromosomes 1/2/6/7/9/10). Chromosomes 1, 2, 7, and 9 each harbored 2 genes, while chromosomes 6 and 10 each contained 1 gene. Among the collinearity relationships of 4 members of the *ZmFAT* family, 2 pairs of duplicated genes were identified (Figure 3). These included *ZmFATA1* and *ZmFATA2*, and *ZmFATB4* and *ZmFATB7*. No collinear gene pairs were found between *ZmFATs* and *AtFATs*, but 1 pair was identified between maize and rice (Figure 4). This result suggests that the differentiation of *ZmFAT*s occurred after the divergence of monocotyledons and dicotyledons.

### 3.5. Cis-Acting Element Analysis

**Figure 5:**
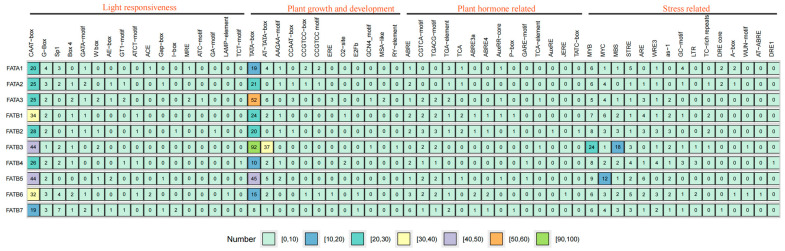
*Cis*-acting elements in promoters. The number of each element is labeled in the corresponding grid. The background color of grids represents the number of elements.

*Cis*-acting element analysis was performed on 2000 bp upstream of *ZmFAT*s obtained from genomic sequences to characterize their potential biological functions (Figure 5). *ZmFAT*s harbored abundant *cis*-acting elements related to light response and plant growth and development. CAAT box and TATA box are the core *cis*-acting elements of *ZmFAT*s. *ZmFATB7* had the fewest CAAT boxes (19) and TATA boxes (8), while *ZmFATB3*/*5* had 44 CAAT boxes. Furthermore, *ZmFATB3* contained the most TATA boxes (92) and AT–TATA boxes (37). In addition, all *ZmFAT* promoters contain 24 light-responsive *cis*-acting elements G box. *ZmFAT*s contain abundant plant hormone-related elements. Except for *ZmFATB3*, other *ZmFAT*s collectively contain 20 ABRE elements. Except for *ZmFATA1*/*2*, other *ZmFAT*s collectively contain 15 CGTCA motifs and 15 TGACG motifs. Numerous *cis*-acting elements of *ZmFAT*s are related to stress response. *ZmFAT*s contain 85 MYB, 40 MYC, and 26 STRE elements. Except for *ZmFATA2*, other *ZmFAT*s contain 33 MBS elements. Except for *ZmFATA1* and *ZmFATB3*, other *ZmFAT*s have 21 ARE elements. Except for *ZmFATB5*, other *ZmFAT*s have 17 WRE3 elements. Except for *ZmFATA1*/*2*, other *ZmFAT*s have 15 as–1 elements. *ZmFATB3* contained the most MYB (24) and MBS (18) elements. *ZmFATB5* had 9 MYB and 12 MYC elements.

### 3.6. Functional Annotation Analysis of ZmFAT Genes

**Figure 6:**
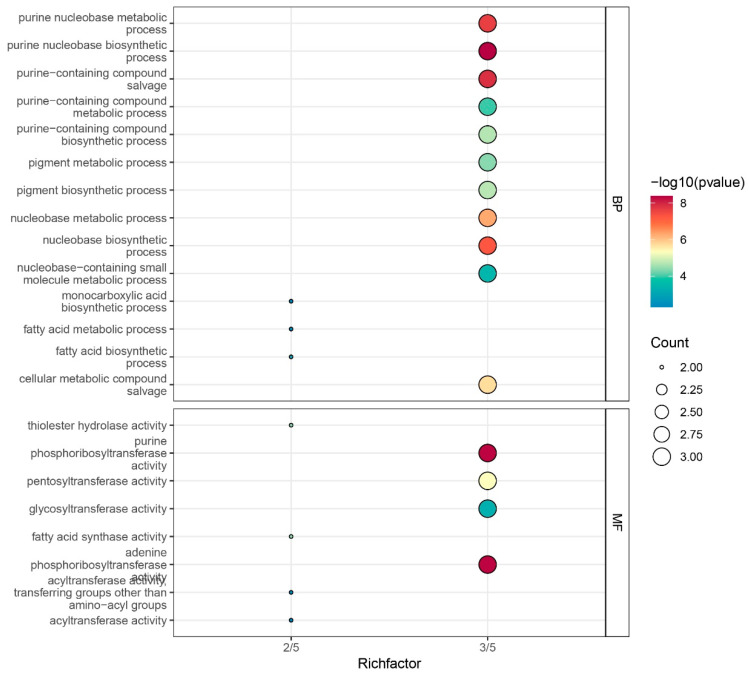
GO annotation analysis of *ZmFAT*s. The horizontal axis represents the proportion of genes annotated with the corresponding functions. The size and color of circles represent the number of genes annotated with functions and −log10 (*p*-value), respectively.

To further investigate the functions of *FAT* genes, GO functional annotation was performed (Table S2). Five *ZmFAT*s were annotated with biological processes and molecular functions (Figure 6). In biological processes, the gene proportions for purine nucleobase metabolic process, purine nucleobase biosynthetic process, purine-containing compound salvage, purine-containing compound metabolic process, purine-containing compound biosynthetic process, pigment metabolic process, pigment biosynthetic process, nucleobase metabolic process, nucleobase biosynthetic process, nucleobase-containing small molecule metabolic process, and cellular metabolic compound salvage were all 3/5. In molecular functions, the proportions for phosphoribosyltransferase activity, pentosyltransferase activity, glycosyltransferase activity, and phosphoribosyltransferase activity were 3/5, indicating their involvement in the formation and conversion of ribose in nucleotides. This suggests that these genes are extensively involved in the synthesis and metabolism of nuclear nucleic acids and their units. Additionally, the proportions for monocarboxylic acid biosynthetic process, fatty acid metabolic process, and fatty acid biosynthetic process were 2/5. The proportions for thiolester hydrolase activity, purine fatty acid synthase activity, adenine transferring groups other than amino–acyl groups, and acyltransferase activity were 2/5. This indicates that *ZmFAT*s participate in fatty acid accumulation by affecting enzyme synthesis.

### 3.7. Interaction Analysis Among ZmFATs and miRNA Regulation Analysis

**Figure 7:**
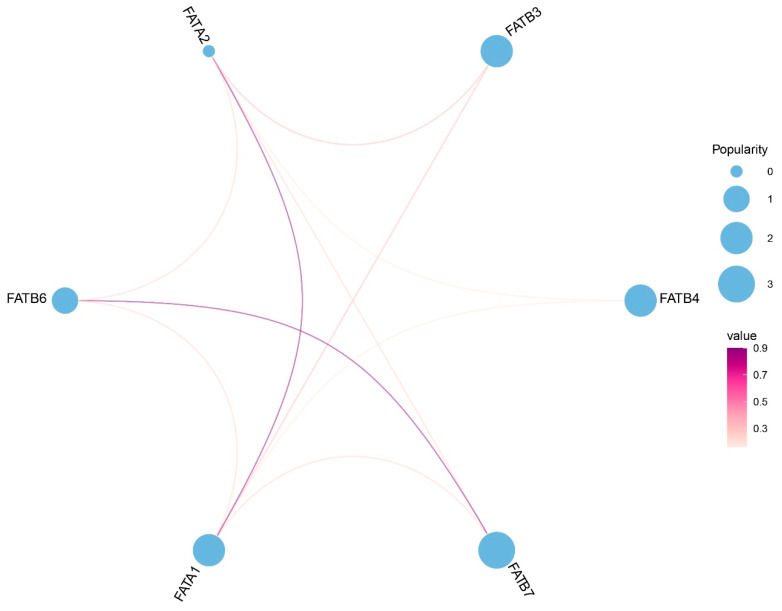
Interaction network of ZmFATs. The size of circles represents popularity. Lines connect genes with correlations. Gradient colors represent the magnitude of correlations.

Protein–protein interaction analysis revealed that six ZmFAT proteins are predicted to interact with each other (Figure 7). Among them, ZmFATA1 and ZmFATA2, as well as ZmFATB6 and ZmFATB7, exhibited the strongest direct interactions, while additional indirect associations were observed between ZmFATA1/2 and ZmFATB3/4/6/7. These results suggest that ZmFATA1 and ZmFATA2 may act as central nodes within the interaction network.

**Figure 8:**
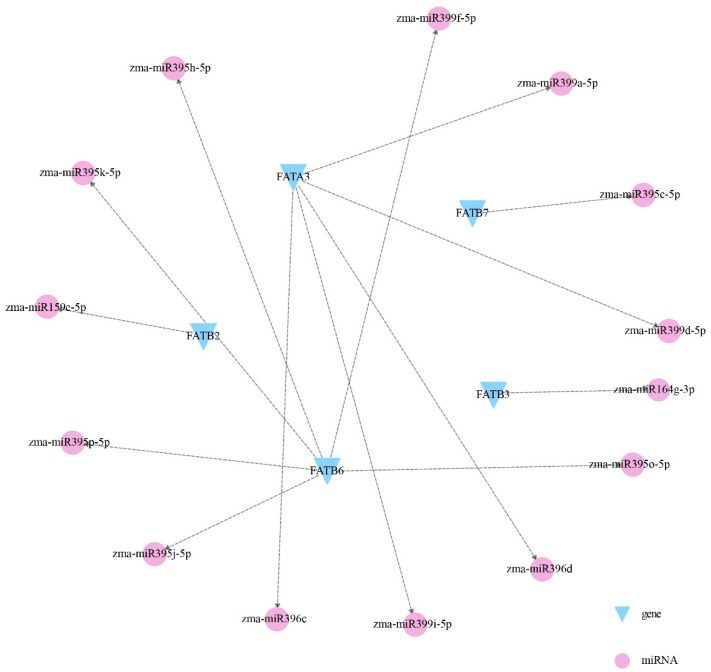
Potential regulation of *ZmFAT*s by miRNAs. Blue triangles represent *ZmFATs*, and purple circles represent miRNAs. miRNAs and *ZmFATs* with correlations are connected by black dashed lines.

Furthermore, gene–miRNA interaction analysis identified 14 miRNAs potentially regulating five *ZmFATs* (Figure 8). Notably, *ZmFATB6* was targeted by the largest number of miRNAs, while five miRNAs regulated *ZmFATA3*. In addition, *ZmFATB2*, *ZmFATB3*, and *ZmFATB7* were regulated by zma–miR159e–5p, zma–miR164g–3p, and zma–miR395c–5p, respectively.

### 3.8. Analysis of ZmFATs Expression Levels in Maize Seeds at Different Developmental Stages

**Figure 9:**
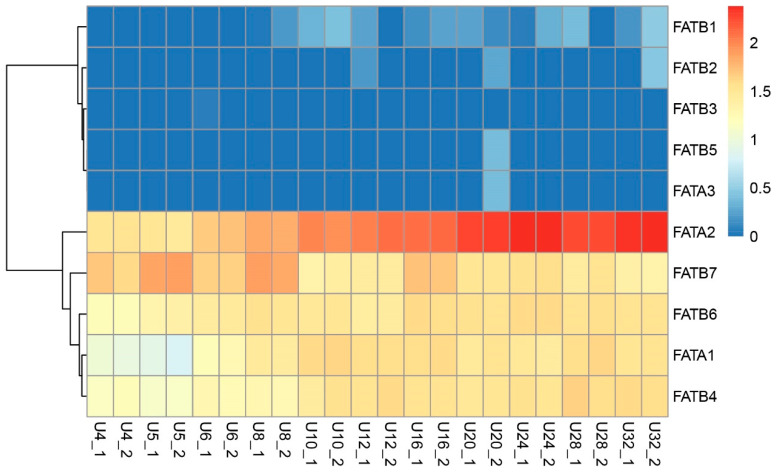
Transcriptomic expression levels of *ZmFAT* genes in the upper tissue (U) of seeds after pollination. The gradient from blue to red represents gradually increasing expression levels. The horizontal axis represents different sampling stages (4, 5, 6, 8, 10, 12, 16, 20, 24, 28, 32 days after pollination).

**Figure 10:**
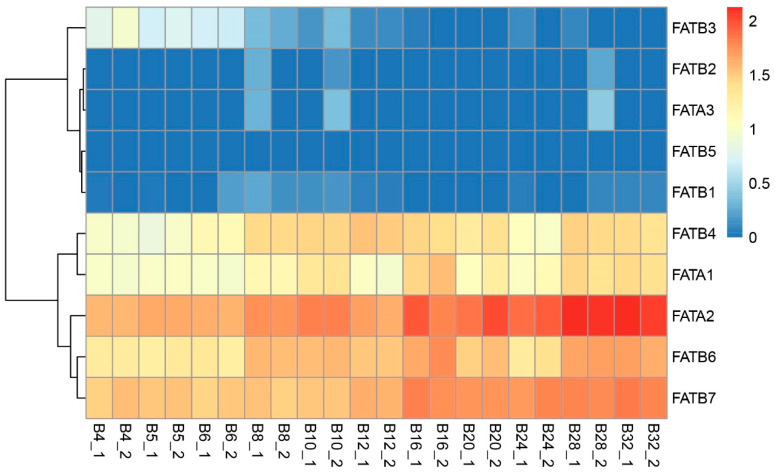
Transcriptomic expression levels of *ZmFAT* genes in the bottom tissue (B) of seeds after pollination. The gradient from blue to red represents gradually increasing expression levels. The horizontal axis represents different sampling stages (4, 5, 6, 8, 10, 12, 16, 20, 24, 28, 32 days after pollination).

Transcriptomic analysis revealed differential expression patterns of ZmFAT genes in maize seeds across developmental stages. Compared with other genes, *ZmFATA1*/*2* and *ZmFATB4*/*6*/*7* consistently exhibited high expression levels during seed development (Figure 9 and Figure 10). During seed development, the expression levels of *ZmFATA1*/*2* and *ZmFATB1*/*4*/*6* in the upper part of maize seeds gradually increased (Figure 9). At later developmental stages, *ZmFAT2* exhibited the highest expression level. *ZmFATB7* showed high expression levels at early developmental stage (especially at 5 d and 8 d), but declined at the later stage. In the lower part of maize seeds, with continuous development, the expression levels of *ZmFATA1*/*2* and *ZmFATB4*/*6*/*7* gradually increased (Figure 10). In the late developmental stage, *ZmFAT2* showed the highest expression level, followed by *ZmFATB6*/*7*. *ZmFATB3* showed high expression levels in the early developmental stage, but expression decreased with continuous development. The expression level of *ZmFATB1* showed fluctuating changes.

**Figure 11:**
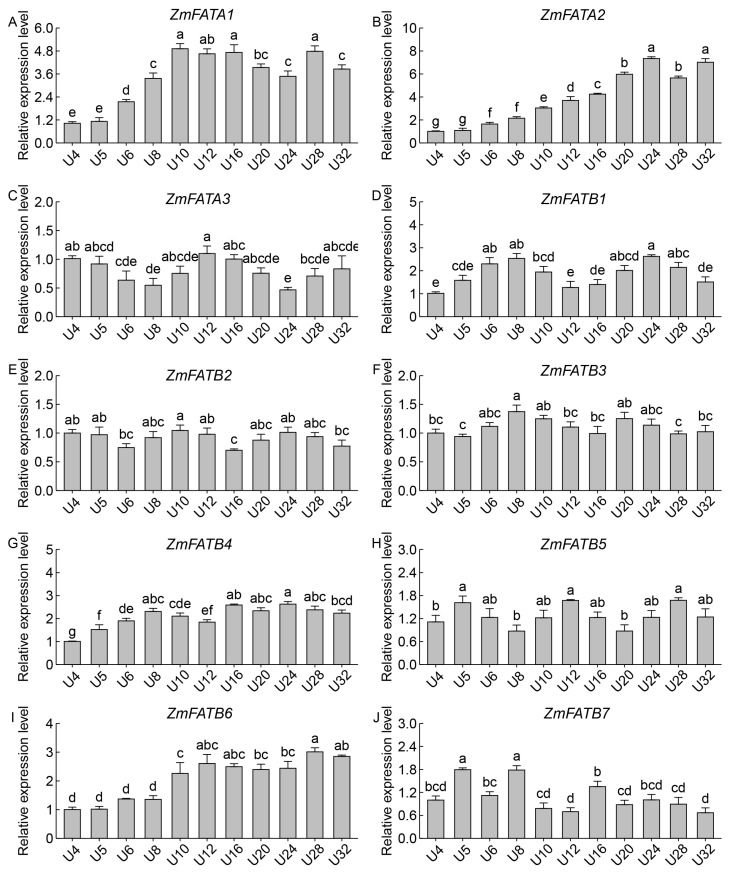
Relative expression levels of *ZmFAT* genes in the upper tissue of seeds after pollination. The horizontal axis represents different sampling stages (4, 5, 6, 8, 10, 12, 16, 20, 24, 28, 32 days after pollination). Different letters indicate significant differences between treatments (*p* < 0.05). (**A**–**J**) represent the expression levels of different genes, with each panel labeled with the corresponding gene name and figure designation.

**Figure 12:**
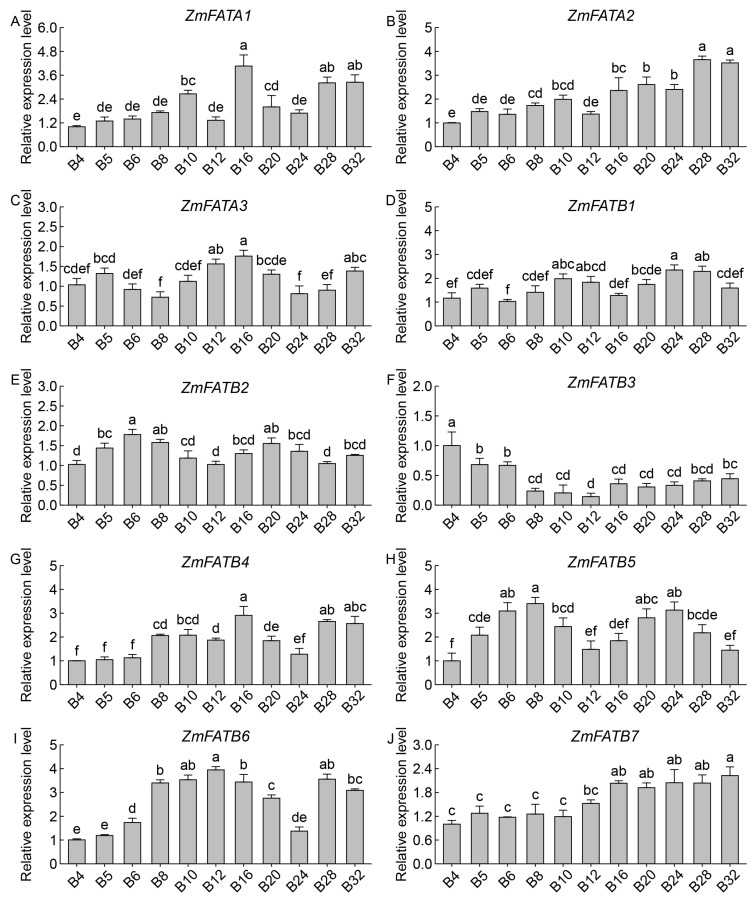
Relative expression levels of *ZmFAT* genes in the lower tissue of seeds after pollination. The horizontal axis represents different sampling stages (4, 5, 6, 8, 10, 12, 16, 20, 24, 28, 32 days after pollination). Different letters indicate significant differences between treatments (*p* < 0.05). (**A**–**J**) represent the expression levels of different genes, with each panel labeled with the corresponding gene name and figure designation.

The qRT–PCR analysis results were similar to the transcriptomic analysis. In both the upper and lower parts of seeds, the expression levels of *ZmFATA1*/*2* and *ZmFATB4*/*6* gradually increased with developmental progression (Figure 11 and Figure 12). *ZmFATB7* had higher expression levels in the early stage of the upper part (Figure 11J) and higher expression levels in the late stage of the lower part (Figure 12J). During seed formation, the expression levels of *ZmFATA3* and *ZmFATB1*/*2*/*5* in both upper and lower parts showed fluctuating changes (Figure 11 and Figure 12). The expression pattern of *ZmFATB3* differed between upper and lower seed regions. In the upper part, *ZmFATB3* expression showed a trend of first increasing then decreasing (Figure 11F). In the lower part, its expression first decreased then slowly increased (Figure 12F). These results indicate that *ZmFAT* genes play important roles in maize seed development, with spatiotemporal differences observed among family members.

## 4. Discussion

Maize oil is characterized by a high proportion of unsaturated fatty acids and a low proportion of saturated fatty acids. These compositional differences from other plant oils may contribute to the favorable nutritional properties of maize oil [39,40]. Differences in fatty acid composition and content are often regulated by FAT enzymes [41]. The availability of the maize genome sequence enabled the identification and characterization of *ZmFAT*s. In this study, 10 *ZmFAT*s sequences were identified from the maize genome. This is similar to studies in *Juglans regia* (8 *FAT* genes) [42] and *Arachis hypogaea* (21 *FAT* genes) [16], indicating that *ZmFATs* is a relatively small gene family. Furthermore, based on amino acid composition and phylogenetic topological analysis, these genes were classified into two subgroups (3 ZmFATAs and 7 ZmFATBs). Moreover, the molecular weights, amino acid compositions, and intron numbers of these *ZmFATs* exhibited considerable variation among members, consistent with observations in other plant species. Such variation may confer functional diversification to *ZmFATs* in maize. In addition, we observed that ZmFATBs were divided into two branches in the phylogenetic tree. This may indicate that ZmFATBs have generated functionally distinct classifications due to divergence.

Collinearity analysis is used to reveal the duplication process of homologous genes. Two pairs of collinear genes were identified. Similarly, two collinear gene pairs were also identified in walnut [43]. Additionally, one collinear gene pair was found between maize and rice. Based on these results, we speculate that *FAT* genes underwent substantial evolutionary changes after the divergence of monocotyledons and dicotyledons. Moreover, this mutation persisted after the divergence of maize and rice.

*Cis*-acting elements provide insights into gene responses under different environmental conditions and stresses signals. CsbZIP50 enhances drought tolerance in cucumber by directly binding to G box/ABRE *cis*-acting elements in the *CsRD29* promoter to activate its expression [44]. MdMYC2 can bind to G box elements in the *MdCBF1* promoter to positively regulate cold tolerance in apple [45]. ABRE *cis*-acting elements play a role in responsive transcription to the stress-related hormone abscisic acid [45]. Overexpression of the *GhGT23* gene can enhance cotton tolerance to salt and drought stress, and this gene can bind to MYB/MBS *cis*-elements to potentially activate expression of downstream resistance genes [46]. In apple, the transcription factors MdMYB9 and MdMYBPA1 bind to MBS elements in the promoters of target genes to activate *MdANS* and *MdUFGT* expression, while mdm-miR858 contributes to anthocyanin accumulation by targeting *MdMYB9* and *MdMYBPA1* [47]. This study found that most *ZmFAT*s contain G box, ABRE, MYB, and MBS *cis*-acting elements. We speculate that *ZmFAT*s play roles in stress resistance in maize by responding to environmental stimuli and hormonal changes. Two ZmFATs possess functions in monocarboxylic acid biosynthetic process, fatty acid metabolic process, fatty acid biosynthetic process, thiolester hydrolase activity, purine fatty acid synthase activity, adenine transferring groups other than amino–acyl groups, and acyltransferase activity. The functional activities of these thiolesterases, acyltransferases, and synthases determine the carbon chain length and proportion of unsaturated fatty acids synthesized in maize [48,49]. This result also underscores the limitation of GO analysis alone, as functional annotation can be incomplete or biased by database curation. Therefore, complementary evidence, including expression profiling and future biochemical validation, will be required to fully elucidate the biological roles of the *ZmFAT* family.

Multiple functional genes cooperate and complement each other and are regulated by multiple different miRNAs [50,51,52]. We found that ZmFATA1/2 interact with multiple other ZmFATs. Moreover, *ZmFATA3* and *ZmFATB6* may be regulated by more miRNAs. We speculate that these genes play more important roles in fatty acid synthesis.

The *ZmFAT* family likely play essential roles in maize seed development by regulating fatty acid metabolic pathways. FATs terminate fatty acid synthesis by hydrolyzing acyl–ACP thioester bonds, releasing free fatty acids and ACP, and providing substrates for subsequent lipid metabolism [7]. This enzyme plays key roles in plant lipid storage and membrane system construction by regulating fatty acid chain length and release rate [8,9,10]. Our results demonstrated that ZmFATA1/2 and ZmFATB4/6/7 consistently exhibited high expression in both the upper and lower parts of maize seeds throughout development (Figure 9 and Figure 10), highlighting their critical functions in lipid storage. Furthermore, we also found that the expression levels of *ZmFATA1*/*2* and *ZmFATB4*/*6* gradually increased with developmental progression (Figure 9, Figure 10, Figure 11 and Figure 12). This may be due to massive lipid accumulation during late seed formation. *ZmFATA7* exhibited opposite temporal dynamic expression characteristics in the upper and lower parts, reflecting its important role in early fat storage in the upper part. Collectively, these findings indicate that *ZmFATA1/2* and *ZmFATB4/6/7* are key genes contributing to lipid storage during maize seed development.

## 5. Conclusions

In this study, a comprehensive genome-wide analysis was performed to characterize the *FAT* gene families in maize, resulting in the identification of 3 *ZmFATA* and 7 *ZmFATB* genes. The *ZmFATs* clustered into different branches in the phylogenetic tree. The gene structures and encoded protein lengths showed considerable differences. *ZmFAT*s contained two pairs of collinear genes within the species. Moreover, as monocotyledonous plants, maize and rice shared one gene pair. The expression of *ZmFAT*s was regulated by light signals and extensively involved in plant growth and development. Furthermore, ZmFATs are potentially involved in the regulation of fatty acid metabolism. Members exhibited interaction relationships and were potentially regulated by 14 miRNAs. *ZmFATA1*/*2* and *ZmFATB4*/*6*/*7* consistently maintained high expression levels in both the upper and lower parts of maize seeds. The expression levels of *ZmFATA1*/*2* and *ZmFATB4*/*6* gradually increased with developmental progression.

## Figures and Tables

**Table 1:** Basic information of maize *FAT* genes.

Protein ID	Name	aa	CDS	MW	pI	GRAVY
Zm00001d006080	*ZmFATA1*	157	471	16,956.17	8.68	−0.17
Zm00001d021046	*ZmFATA2*	203	609	22,119.05	8.24	−0.14
Zm00001d026569	*ZmFATA3*	426	1278	47,666.77	6.37	−0.34
Zm00001d029901	*ZmFATB1*	160	480	17,915.04	5.4	−0.54
Zm00001d029902	*ZmFATB2*	142	426	16,029.88	8.64	−0.75
Zm00001d005148	*ZmFATB3*	379	1137	42,303.49	9.19	−0.44
Zm00001d036137	*ZmFATB4*	239	717	25,933.46	8.56	−0.08
Zm00001d021004	*ZmFATB5*	83	249	9737.42	10.29	−0.37
Zm00001d045387	*ZmFATB6*	434	1302	47,759.83	9.01	−0.39
Zm00001d046454	*ZmFATB7*	418	1254	46,396.3	8.25	−0.36

## Supplementary Materials

The following supporting information can be downloaded at: https://www.mdpi.com/article/10.3390/genes16091035/s1, Table S1: The primer sequences of *FAT* genes; Table S2: GO functional annotation.

## References

[1] Xu Y., Singer S.D., Chen G. (2023). Protein interactomes for plant lipid biosynthesis and their biotechnological applications. Plant Biotechnol. J..

[2] Jump D.B. (2004). Fatty acid regulation of gene transcription. Crit. Rev. Clin. Lab. Sci..

[3] Guan X., Okazaki Y., Zhang R., Saito K., Nikolau B.J. (2020). Dual-localized enzymatic components constitute the fatty acid synthase systems in mitochondria and plastids. Plant Physiol..

[4] Ashton A.R., Jenkins C.L., Whitfeld P.R. (1994). Molecular cloning of two different cDNAs for maize acetyl CoA carboxylase. Plant Mol. Biol..

[5] Kremer L., Nampoothiri K.M., Lesjean S., Dover L.G., Graham S., Betts J., Brennan P.J., Minnikin D.E., Locht C., Besra G.S. (2001). Biochemical characterization of acyl carrier protein (AcpM) and malonyl-CoA: AcpM transacylase (MtFabD), two major components of *Mycobacterium tuberculosis* fatty acid synthase II. J. Biol. Chem..

[6] Zhang S., Wu S., Hou Q., Zhao J., Fang C., An X., Wan X. (2024). Fatty acid de novo biosynthesis in plastids: Key enzymes and their critical roles for male reproduction and other processes in plants. Plant Physiol. Biochem..

[7] Aznar-Moreno J.A., Sánchez R., Gidda S.K., Martínez-Force E., Moreno-Pérez A.J., Venegas Calerón M., Garcés R., Mullen R.T., Salas J.J. (2018). New insights into sunflower (*Helianthus annuus* L.) FatA and FatB thioesterases, their regulation, structure and distribution. Front. Plant Sci..

[8] Bates P.D., Shockey J. (2025). Towards rational control of seed oil composition: Dissecting cellular organization and flux control of lipid metabolism. Plant Physiol..

[9] Dehesh K., Tai H., Edwards P., Byrne J., Jaworski J.G. (2001). Overexpression of 3-ketoacyl-acyl-carrier protein synthase IIIs in plants reduces the rate of lipid synthesis. Plant Physiol..

[10] Higashi Y., Saito K. (2019). Lipidomic studies of membrane glycerolipids in plant leaves under heat stress. Prog. Lipid Res..

[11] Feng Y., Wang Y., Liu J., Liu Y., Cao X., Xue S. (2017). Structural insight into acyl-ACP thioesterase toward substrate specificity design. ACS Chem. Biol..

[12] Bahadır S., Abdulla M.F., Mostafa K., Kavas M., Hacıkamiloğlu S., Kurt O., Yıldırım K. (2024). Exploring the role of FAT genes in Solanaceae species through genome-wide analysis and genome editing. Plant Genome.

[13] Salas J.J., Ohlrogge J.B. (2002). Characterization of substrate specificity of plant FatA and FatB acyl-ACP thioesterases. Arch. Biochem. Biophys..

[14] Zhou Z., Zhang D., Lu M. (2007). Cloning and expression analysis of *PtFATB* gene encoding the acyl-acyl carrier protein thioesterase in *Populus tomentosa* Carr. J. Genet. Genom..

[15] Zhou Z., Lakhssassi N., Knizia D., Cullen M.A., El Baz A., Embaby M.G., Liu S., Badad O., Vuong T.D., AbuGhazaleh A. (2021). Genome-wide identification and analysis of soybean acyl-ACP thioesterase gene family reveals the role of GmFAT to improve fatty acid composition in soybean seed. Theor. Appl. Genet..

[16] Peng Z., Zhang H., Tian H., Shan L., Zhang Z., Ding H., Gao W., Li X. (2020). The phylogeny and functional characterization of peanut acyl-ACP Thioesterases. J. Plant Growth Regul..

[17] Jones A., Davies H.M., Voelker T.A. (1995). Palmitoyl-acyl carrier protein (ACP) thioesterase and the evolutionary origin of plant acyl-ACP thioesterases. Plant Cell.

[18] Nam J.-W., Yeon J., Jeong J., Cho E., Kim H.B., Hur Y., Lee K.-R., Yi H. (2019). Overexpression of acyl-ACP thioesterases, *CpFatB4* and *CpFatB5*, induce distinct gene expression reprogramming in developing seeds of *Brassica napus*. Int. J. Mol. Sci..

[19] Lin N., Ai T., Gao J., Fan L., Wang S., Chen F. (2013). Identification of novel acyl-ACP thioesterase gene ClFATB1 from *Cinnamomum longepaniculatum*. Biochemistry.

[20] Aznar-Moreno J.A., Venegas-Calerón M., Martínez-Force E., Garcés R., Salas J.J. (2016). Acyl carrier proteins from sunflower (*Helianthus annuus* L.) seeds and their influence on FatA and FatB acyl-ACP thioesterase activities. Planta.

[21] Liu B., Sun Y., Wang X., Xue J., Wang J., Jia X., Li R. (2022). Identification and functional characterization of acyl-ACP thioesterases B (GhFatBs) responsible for palmitic acid accumulation in cotton seeds. Int. J. Mol. Sci..

[22] Dormann P., Voelker T.A., Ohlrogge J.B. (2000). Accumulation of palmitate in *Arabidopsis* mediated by the acyl-acyl carrier protein thioesterase FATB1. Plant Physiol..

[23] Han H., Wu W., Hou H., Zhang M., Guo A., Zhou Y., Liu J., Li K., Bai S., Li B. (2024). Function analysis of transcription factor OSR1 regulating osmotic stress resistance in maize. Biochem. Biophys. Res. Commun..

[24] Stamenković O.S., Kostić M.D., Tasić M.B., Djalović I.G., Mitrović P.M., Biberdžić M.O., Veljković V.B. (2020). Kinetic, thermodynamic and optimization study of the corn germ oil extraction process. Food Bioprod. Process..

[25] Maki K., Dicklin M., Cassens M., Bell M., Bunczek M., Eren F. (2019). Predictors of Cholesterol Lowering with Corn Oil Consumption: Results from a Pooled Analysis of Randomized, Free-living Feeding Trials (P08-112-19). Curr. Dev. Nutr..

[26] Wang J.-K., Li Y., Zhao X.-L., Liu Y.-B., Tan J., Xing Y.-Y., Adi D., Wang Y.-T., Fu Z.-Y., Ma Y.-T. (2022). Ablation of plasma prekallikrein decreases low-density lipoprotein cholesterol by stabilizing low-density lipoprotein receptor and protects against atherosclerosis. Circulation.

[27] Marchler-Bauer A., Derbyshire M.K., Gonzales N.R., Lu S., Chitsaz F., Geer L.Y., Geer R.C., He J., Gwadz M., Hurwitz D.I. (2015). CDD: NCBI’s conserved domain database. Nucleic Acids Res..

[28] Lei B., Song M., Li X., Dang X., Qin R., Zhu S., An X., Liu Q., Yao X., Nie Y. (2022). SMART V1.0: A database for small molecules with functional implications in plants. Interdiscip. Sci. Comput. Life Sci..

[29] Duvaud S., Gabella C., Lisacek F., Stockinger H., Ioannidis V., Durinx C. (2021). Expasy, the Swiss Bioinformatics Resource Portal, as designed by its users. Nucleic Acids Res..

[30] Kumar S., Stecher G., Tamura K. (2016). MEGA7: Molecular evolutionary genetics analysis version 7.0 for bigger datasets. Mol. Biol. Evol..

[31] Bailey T.L., Johnson J., Grant C.E., Noble W.S. (2015). The MEME suite. Nucleic Acids Res..

[32] Chen C., Chen H., Zhang Y., Thomas H.R., Frank M.H., He Y., Xia R. (2020). TBtools: An integrative toolkit developed for interactive analyses of big biological data. Mol. Plant.

[33] Wang Y., Tang H., Wang X., Sun Y., Joseph P.V., Paterson A.H. (2024). Detection of colinear blocks and synteny and evolutionary analyses based on utilization of MCScanX. Nat. Protoc..

[34] Rombauts S., Déhais P., Van Montagu M., Rouzé P. (1999). PlantCARE, a plant cis-acting regulatory element database. Nucleic Acids Res..

[35] Szklarczyk D., Nastou K., Koutrouli M., Kirsch R., Mehryary F., Hachilif R., Hu D., Peluso M.E., Huang Q., Fang T. (2025). The STRING database in 2025: Protein networks with directionality of regulation. Nucleic Acids Res..

[36] Singhal A., Cao S., Churas C., Pratt D., Fortunato S., Zheng F., Ideker T. (2020). Multiscale community detection in Cytoscape. PLoS Comput. Biol..

[37] Farid B., Saddique M.A.B., Tahir M.H.N., Ikram R.M., Ali Z., Akbar W. (2025). Expression divergence of BAG gene family in maize under heat stress. BMC Plant Biol..

[38] Qian B., Wang Q., Zhang C., Guo J., Yu Z., Han J., Xia H., Zhao R., Yin Y. (2024). Exploring the Roles of TALE Gene Family in Maize Drought Stress Responses. Agronomy.

[39] Hall J.A., Van Saun R.J., Tornquist S.J., Gradin J.L., Pearson E.G., Wander R.C. (2004). Effect of type of dietary polyunsaturated fatty acid supplement (corn oil or fish oil) on immune responses in healthy horses. J. Vet. Intern. Med..

[40] Apgar J.L., Shively C.A., Tarka Jr S.M. (1987). Digestibility of cocoa butter and corn oil and their influence on fatty acid distribution in rats. J. Nutr..

[41] Tan K.W.M., Lee Y.K. (2017). Expression of the heterologous Dunaliella tertiolecta fatty acyl-ACP thioesterase leads to increased lipid production in *Chlamydomonas reinhardtii*. J. Biotechnol..

[42] Wang H., Shi J., Guo W., Sun X., Niu S., Chen L., Liu S., Ma L. (2024). The identification and expression analysis of walnut Acyl-ACP thioesterases. Front. Genet..

[43] Li Y., Xian X., Guo L., Zhang J., Gan C., Wang Z., Li H., Li X., Yuan X., Zhang N. (2022). CsbZIP50 binds to the G-box/ABRE motif in CsRD29A promoter to enhance drought tolerance in cucumber. Environ. Exp. Bot..

[44] Wang Y., Xu H., Liu W., Wang N., Qu C., Jiang S., Fang H., Zhang Z., Chen X. (2019). Methyl jasmonate enhances apple’cold tolerance through the JAZ–MYC2 pathway. Plant Cell Tissue Organ Cult. (PCTOC).

[45] Soma F., Takahashi F., Yamaguchi-Shinozaki K., Shinozaki K. (2021). Cellular phosphorylation signaling and gene expression in drought stress responses: ABA-dependent and ABA-independent regulatory systems. Plants.

[46] Li Y., Hu Z., Dong Y., Xie Z. (2023). Overexpression of the cotton trihelix transcription factor *GhGT23* in *Arabidopsis* mediates salt and drought stress tolerance by binding to GT and MYB promoter elements in stress-related genes. Front. Plant Sci..

[47] Li Z., Liu W., Chen Q., Zhang S., Mei Z., Yu L., Wang C., Mao Z., Chen Z., Chen X. (2023). Mdm-miR858 targets *MdMYB9* and *MdMYBPA1* to participate anthocyanin biosynthesis in red-fleshed apple. Plant J..

[48] He M., Qin C.-X., Wang X., Ding N.-Z. (2020). Plant unsaturated fatty acids: Biosynthesis and regulation. Front. Plant Sci..

[49] Thelen J.J., Ohlrogge J.B. (2002). Metabolic engineering of fatty acid biosynthesis in plants. Metab. Eng..

[50] Wang Y., Shen Y., Dong W., Cai X., Yang J., Chen Y., Jia B., Sun M., Sun X. (2024). PHD17 acts as a target of *miR1320* to negatively control cold tolerance via JA-activated signaling in rice. Crop J..

[51] Megha S., Basu U., Kav N.N. (2018). Regulation of low temperature stress in plants by microRNAs. Plant Cell Environ..

[52] Zhang Q., Shen L., Ren D., Hu J., Chen G., Zhu L., Gao Z., Zhang G., Guo L., Zeng D. (2019). Characterization, expression, and interaction analyses of *OsMORF* gene family in rice. Genes.

