# Supplementary material for: Comprehensive Characterization of the FATs Gene Family in Maize: Phylogeny, Expression Patterns, and Regulatory Networks
Source: Genes (Basel). 2025 Aug 30;16(9):1035. doi: 10.3390/genes16091035 (PMC12470007; doi:10.3390/genes16091035)
Supplement: Supplementary file 1 [file genes-16-01035-s001.zip › FAT_Table S1.pdf]

Table S1: The primer sequences of *FAT* genes.

Primer	**Sequence**
ZmActin1-F	TACGAGATGCCTGATGGTCAGGTCA
ZmActin1-R	TGGAGTTGTACGTGGCCTCATGGAC
ZmFATA1-F	CTGGAGGACGGGCTCTCTT
ZmFATA1-R	AGTAGTTGTGGCGAAGCCATC
ZmFATA2-F	TTGGGTTCTCCACTGATGGC
ZmFATA2-R	GAGGATCCAATCACGACGGG
ZmFATA3-F	TGGCTCAAGCACAAGAATACCT
ZmFATA3-R	GACAGATGGACTGCACGACA
ZmFATB1-F	CCTTTGGCGAGCAAAACACA
ZmFATB1-R	CCATCAATGCCTACGCTCCT
ZmFATB2-F	TTCCAGGAAGCGAGGAATGG
ZmFATB2-R	TGTCGCCTTCAGTGTTGTGT
ZmFATB3-F	AATAATGGCAGGCGTGTTGC
ZmFATB3-R	TCCCTTCCGCAAACTTACCC
ZmFATB4-F	GTGCCGATCGTACTGCTTCT
ZmFATB4-R	GGTTTGCGGAAACGTACCAC
ZmFATB5-F	CGGAACTTGTTCTGGGTGGT
ZmFATB5-R	GCCTTCAATATTGTGTGGCCT
ZmFATB6-F	ACGAAAACTCTGGACGCACA
ZmFATB6-R	CATAGTACCACCGTCAGGAGC
ZmFATB7-F	GATTGGGGCAGATAGGACGG
ZmFATB7-R	CGATGATGGCCTGCATTTGG
